# Latent profile analysis of chronic physical illness effects on psychiatric treatment outcomes in severe mental disorders

**DOI:** 10.1192/j.eurpsy.2026.12153

**Published:** 2026-07-31

**Authors:** K. Matić, K. Bosak, M. Grah, D. Bodor, N. Mayer, V. Grošić, Ž. Bajić, I. Simunovic Filipcic, I. Filipčić

**Affiliations:** 1 University Psychiatric Clinic Sveti Ivan, Zagreb; 2 Faculty of Dental Medicine and Health, Josip Juraj Strossmayer University of Osijek, Osijek; 3 University of Applied Health Science; 4 Department of psychiatry and psychological medicine, University Hospital Centre Zagreb, Zagreb, Croatia

## Abstract

**Introduction:**

Chronic physical illnesses (CPIs) are common in severe mental disorders (SMD) and may shape psychiatric trajectories, yet heterogeneity across multimorbidity patterns is rarely modelled explicitly. We used latent profile analysis (LPA) to identify CPI profiles and examined their association with treatment outcomes.

**Objectives:**

To derive CPI-based latent profiles in SMD and compare psychiatric rehospitalisations and related outcomes across profiles.

**Methods:**

We conducted a retrospective cohort study on 605 patients with SMD. Eighteen binary CPI indicators (European Health Interview Survey) were analysed. Principal component analysis on tetrachoric correlations yielded five CPI components (musculoskeletal; cardiovascular; respiratory–metabolic; metabolic/general health; cerebrovascular). LPA was then fitted to these component scores, comparing four variance–covariance structures and 1–8 classes. The optimal solution was chosen using information criteria, entropy and clinical interpretability. Profiles were compared on the number of rehospitalisations for relapse (primary endpoint) using robust MM-estimation, adjusting for age, sex, education, household income, disorder duration, number of other psychiatric diagnoses, and treatment with antipsychotics, benzodiazepines, mood stabilisers and antidepressants.

**Results:**

We included 605 patients: 326 schizophrenia-spectrum, 223 recurrent major depressive disorder (moderate–severe) and 56 bipolar affective disorder. A four-profile solution provided the best fit and clinical coherence: (1) healthiest/low multimorbidity (mean 1.1 CPI); (2) cerebrovascular–metabolic (2.5 CPI); (3) cardiometabolic–musculoskeletal (3.8 CPI); (4) high-burden musculoskeletal/general health with multiple comorbidities (5.1 CPI). Rehospitalisations differed across profiles: the highest burden occurred in Profile 3, Profiles 1 and 4 showed lower counts, with Profile 2 intermediate; patterns persisted after covariate adjustment.

**Conclusions:**

LPA identified clinically distinct multimorbidity profiles in SMD that were differentially associated with psychiatric rehospitalisations for relapse. Patients with combined cardiometabolic–musculoskeletal burden had the poorest course, whereas low-burden and concentrated high-risk profiles fared better. CPI profiling may support risk stratification and tailored integrated somatic–psychiatric care and inform service planning.

**Disclosure of Interest:**

None Declared

